# Professor Nirmal Surya, Neurologist at Bombay Hospital & Saifee Hospital, Mumbai, India: Adapted Interview from the 12^th^ World Congress for NeuroRehabilitation (WCNR), Vienna, 2022

**DOI:** 10.25122/jml-2023-1006

**Published:** 2023-02

**Authors:** Andreea Doria Constantinescu, Alexandra Gherman

**Affiliations:** 1 RoNeuro Institute for Neurological Research and Diagnostic, Cluj-Napoca, Romania

**Interviewer: Andreea Doria Constantinescu**

**Interviewee: Professor Nirmal Surya**

Professor Nirmal Surya (DNB, MD, MBBS) is the Past President of the Indian Academy of Neurology, Founder President of the Indian Federation of Neurorehabilitation, and Founder Trustee & Chairman of the Epilepsy Foundation, Mumbai, India. With over 30 years of experience, his areas of expertise include neurorehabilitation, stroke, and epilepsy.

Professor Surya has attended a large number of seminars, workshops, conferences, and symposia in the field of neurology & neurorehabilitation.

**D.A.C.:** Dear Professor Nirmal Surya, we are here, in Vienna, for the 12^th^ World Congress for NeuroRehabilitation, organised by the World Federation for NeuroRehabilitation. What is your first-hand opinion of the event so far, and have you participated in any previous editions?

**N.S.:** I have been associated with the WFNR and WCNR for a long time. In 1996 it was the first world congress on neurorehabilitation, and I was there and presented the poster; then, from 2006 Hong Kong onward, I had been regularly presenting, participating, actively involved. In fact, in 2018 – Mumbai, the last physical conference was organised by me; it was the most successful when more than 2,000 participants were there from 56 countries. What I gather today, after 3 years of Covid lockdown is that we can see that there is a buzz, people want to come, people are happy, they are happy to meet, and so far, at the discussions and the presentations, I found that all the halls are busy and it is all about neurotechnology, neurorehabilitation, a newer technology that people had been talking about and I think that whatever changes happened in the last three, four years, [these] have been discussed. I think this is a change which has come because, for so many years, people have been stuck in lockdown, and could not travel because of Covid; now they find it like a sort of outlet and are very happy.

**D.A.C.:** What is the overarching theme of the congress this year, from your point of view?

**N.S.:** I think the theme would be future technologies because what I gathered today from the plenary lectures and other lectures is that we are more focused on neuro-technology, we are talking about the brain-computer interfaces (BCI), we are talking about the technology, and nano-technology, so I think it is the neuro-technology in neurorehabilitation, but we can talk about Rehabilitation 2030.

**D.A.C.:** From your perspective, what is the role of hybrid multidisciplinary events in developing neurorehabilitation research and practice, and what similar avenues are worth exploring?

**N.S.:** I feel that the hybrid mode of conferencing is the norm now. If you look at all the conferences that happened in the last year or six months since we started, there was that of the American Academy, the European Academy, and the American Epilepsy Association; they were all hybrid. Hybrid gives opportunity to those people who cannot travel. Because now, there is a challenge: post-Covid; travelling is not easy, and the costs are enormous, particularly for people who are coming from developing countries – they find it hard to travel. The air travel costs, the hotel costs, they are 3 times or 4 times higher. Therefore, the hybrid meeting gives an opportunity to people who are really interested in learning to join and learn online.

**D.A.C.:** It is great coverage, in this way, to reach all the interested participants.

**N.S.:** That is correct!

**D.A.C.:** What is the level of access to neurorehabilitation facilities and educational programmes in Asian countries?

**N.S.:** In Asia, there are developed countries, like Japan, Korea, and Hong Kong, and there are many non-developed countries or developing countries. There is a great disparity in the neurorehabilitation specialists present in developing countries; they are not technologically very, very smart concerning robotics, and all these neuro-technologies are not available in developing countries. There are some countries where neurorehabilitation is very scanty or very few specialists are available. And therefore, it is very important to develop neurorehabilitation in Asia; we really need to come up with a plan where we can increase neurorehabilitation expertise and create an education. IFNR in India, in the last years of lockdown, for 4 years onwards, has been doing a lot of educational programmes; we have done more than 100 webinars, and people from all over the world have attended. We know that there is a great need for education and training, and we will continue to put our efforts in this direction.

**D.A.C.:** How important is the role of the community in providing a proper long-term follow-up of the stroke patient?

**N.S.:** The community and the family are, and let us not forget about the families. I have always talked about family-based rehabilitation. Now, post-Covid, the whole scenario has changed: community, family, and telerehabilitation. If you combine all these, then, particularly for the people who come from nuclear families or are staying in the metro-cities, where they don’t have a big community together or family together, it gives them the opportunity to provide long-term rehabilitation. In my opinion, the future will be a combination of community-based rehabilitation, plus/minus family-based rehabilitation, to which telerehabilitation is added. If these work, we can provide low costs, and regular therapy, even for the stroke patient who cannot afford to go and travel for a long period.

**D.A.C.:** What are the immediate needs of the Indian medical system to implement increased awareness and rehabilitation for stroke patients?

**N.S.:** In India, we are growing; more people are getting trained, and we need to develop low-cost rehab centres. We need to have systematic training programmes for stroke rehabilitation. The neurologists need to take a lead – there are centres which are highly advanced, with RDMS (Registered Diagnostic Medical Sonographer) and GCDS, but there are centres where nothing is available. We really need to bring a minimum standard therapy and the need for neuropsychologists and swallowing assessments. We need to gear the stroke specialists to assess swallowing right on day 1, which can prevent a lot of complications later in the case of stroke. These are the needs which I think we need to address faster.

**D.A.C.:** How efficient is yoga in reducing the impact of epilepsy? Is this form of treatment embraced by the patients?

**N.S.:** This is a double-blind trial which we started on yoga and epilepsy. One of my patients with epilepsy is a young girl who does yoga. I told her: "You are suffering from epilepsy; you do yoga, you are better; you do the training for other epilepsy patients". We are doing a double-blind, randomized control trial with 50 patients – 25 with epilepsy and 25 with epilepsy well-controlled. And we find that yoga helps. It is all about the well-being of the patient; it may not be directly impacting epilepsy, and therefore, it is very important that you take the medicine, you do regular yoga so that the associated depression, the stress which can precipitate your attack, can be reduced. Yoga is not only for epilepsy. We have yoga for Parkinson’s disease, we have yoga for multiple sclerosis, and we have yoga for stroke. We have devised certain yoga from ancient end-system which can be used for these diseases, and we have done a proper study of Parkinson’s disease and yoga, and it has helped Parkinson’s disease patients.

**D.A.C.:** This is very interesting!

**N.S.:** Thank you!

**D.A.C.:** What could be some national strategies for mediating Covid-related anxiety in people with epilepsy?

**N.S.:** We have done a study on Covid and anxiety. Fortunately, and unfortunately, we did not get many people with epilepsy to have the Covid. Number 1. Number 2 – when we assessed them on Covid, pre- and post-, even the before Covid assessment, and during the Covid and after the Covid assessment, there was not much change. Except for a mild increase in anxiety. I think that as long as my patients knew the doctor was available to them – (I was available through the lockdown, online or offline), their anxiety level was not increased. The only challenges came when the medicine was not available because of the lockdown, the medical shops were closed, and they did not have the prescriptions. That was a challenge; they had that fear to get an attack (without medicine) then. Once those things settled down, I think they were alright.

**D.A.C.:** Thank you very much for your time and for being here today with us!

**N.S.:** Thank you for the interview, and I hope it gives information to the people and they like it.

